# Cardiovascular Health and The Intestinal Microbial Ecosystem: The Impact of Cardiovascular Therapies on The Gut Microbiota

**DOI:** 10.3390/microorganisms9102013

**Published:** 2021-09-23

**Authors:** Noora Alhajri, Rubiya Khursheed, Mohammad Taher Ali, Tareq Abu Izneid, Oumaima Al-Kabbani, Mahdia B. Al-Haidar, Fatima Al-Hemeiri, Mohamed Alhashmi, Faheem Hyder Pottoo

**Affiliations:** 1 Department of Epidemiology and Population Health, College of Medicine and Health Sciences, Khalifa University, Abu Dhabi P.O. Box 127788, United Arab Emirates; 100058271@ku.ac.ae (O.A.-K.); 100053510@ku.ac.ae (M.B.A.-H.); 100053253@ku.ac.ae (F.A.-H.); 100053507@ku.ac.ae (M.A.); 2 Department of Medicine, Sheikh Shakhbout Medical City (SSMC), Abu Dhabi P.O. Box 11001, United Arab Emirates; 3 Department of Pharmaceutical Sciences, Lovely Professional University, Punjab 144403, India; rubiyakhursheed141@gmail.com; 4 Department of Pharmacology, College of Clinical Pharmacy, Imam Abdulrahman Bin Faisal University, P. O. Box 1982, Dammam 31441, Saudi Arabia; mtali@iau.edu.sa (M.T.A.); fhpottoo@iau.edu.sa (F.H.P.); 5 Pharmaceutical Sciences, College of Pharmacy, Al Ain University, Al Ain, Abu Dhabi P.O. Box 112612, United Arab Emirates; tareq.abuizneid@aau.ac.ae

**Keywords:** dysbiosis, Firmicutes, Bacteroidetes, cardiovascular diseases, coronary artery disease, hypertension, heart failure, stroke, peripheral vascular disease, rheumatic heart disease, cardiomyopathies

## Abstract

It has become evident over the past several years that the intestinal microbial ecosystem plays a critical role in the development and prevention of cardiovascular diseases (CVDs) and other metabolic disorders, such as hypertension, obesity, diabetes mellitus, and metabolic syndrome. The intestinal microbiota ecosystem functions as a major virtual endocrine organ that interacts and responds to molecules’ signals within the host. Several meta-organismal pathways are involved in the gut–host interaction, including trimethylamine-N-oxide (TMAO) and short-chain fatty acids (SCFA). Host phenotype and cardiovascular diseases (CVDs) varying from hypertension, insulin resistance, and obesity to more specific inflammatory processes, such as atherosclerosis and hypercoagulability, have shown to be affected by the gut–host interaction. Additionally, several studies that involved animals and humans demonstrated a striking connection between the development of new CVDs and an imbalance in the gut microbiota composition along with the presence of their derived metabolites. Through this review article, we aim to evaluate the role of the normal gut microbiota ecosystem, its association with CVDs, effects of the therapies used to control and manage CVDs in the gut microbiota environment and explore potential therapeutic interventions to amplify disease outcomes in patients with CVDs.

## 1. Introduction

Cardiovascular diseases (CVDs) can refer to several conditions, including hypertension, heart failure, stroke, coronary artery disease, peripheral vascular disease, stroke, rheumatic heart disease, cardiomyopathies, and congenital heart diseases [1]. Globally, CVDs are major contributors to a decreased quality of life and the principal cause of morbidity and mortality. In 2015, 422.7 million cases of CVDs were reported, with 17.92 million deaths due to CVDs. Of all the causes, coronary heart disease was the major cause of CVDs worldwide [2]. As far as Middle Eastern countries are concerned, CVDs are responsible for 34% of all deaths, with coronary artery disease contributing to 44% of CVDs, followed by stroke with 35% of CVDs [3,4].

Hypertension (which is defined as systolic BP ≥130 mmHg and/or diastolic BP ≥80 mmHg) [5] is the third leading cause of years lost due to morbidity-related disabilities. It increases the risk of heart, kidney, brain, and other diseases. It can be easily diagnosed, treated, and controlled, thus reducing the morbidity and mortality. Globally, it is estimated to affect 1.13 billion people, with two-thirds of them living in developing countries. The global target is to reduce its prevalence by 25% by 2025. Globally, hypertension alone is responsible for 54% of stroke, 47% of coronary artery disease, and 13.5% of the total premature deaths (7.6 million) worldwide [6]. Heart failure (HF) is a disease that occurs due to a low cardiac output or an elevated ventricular-filling pressure with increasing prevalence; it is considered a global pandemic, affecting as many as 26 million people [7]. Data showing the prevalence of heart failure and associated mortality rate are deficient in Middle Eastern countries. However, a recent study published in Saudi Arabia showed the overall 30-day mortality rate for 1090 acute HF patients to be 7.5%. In the Sultanate of Oman, the prevalence of heart failure is 5.17 per 1000 individuals, which seems to be less than the prevalence recorded in some of the developed countries. Apparently, the analysis of the data was taken from a single center. However, over the last decade, improved diagnostic methods, such as imaging, and the availability of new diagnostic indicators, therapeutic advances, and implantable devices have helped to delay the deaths of heart failure patients [8].

Atherosclerosis is a major cause of CVDs. It is due to the progressive inflammation of the arteries that is characterized by focal subintimal deposition of fat that remains subclinical until it progresses in size and impairs tissue perfusion or an underlying atheromatous lesion that ulcerates and disrupts, resulting in the formation of thrombotic occlusion or distal embolization of the vessel. Its common risk factors include cigarette smoking, hypercholesterolemia, hypertension, and diabetes mellitus. These risk factors, along with immunologic phenomenon, inflammation, and endothelial dysfunction, trigger the formation of fatty streaks in the arterial walls, which give rise to the characteristic plaque formation that may develop as early as during early childhood. The plaque consists of an accumulation of lipid-laden foam cells in the tunica intima and extracellular matrix with the proliferation of smooth muscle cells. The rupture of the plaque can eventually lead to arterial thrombosis, causing coronary artery disease or a stroke. As the disease progresses, the CRP (C-reactive protein), CD40, and the cardiac myofilament protein troponin can be detected as early indicators of acute coronary syndromes. Atherosclerosis of the coronary arteries is a major cause of heart failure. Abnormal calcium signaling to the myofilaments also appears to be responsible for the development of HF and cardiomyopathy. Uncontrolled hypertension can result in left ventricular hypertrophy through biochemical and neurohumoral mechanisms, which may cause heart failure [9].

Recently, several studies have found that intestinal microbiota plays a critical role in the development of CVDs [10]. The term “Intestinal microbiota”’ is commonly used for various groups of microorganisms that are living within the human digestive tract and help the host by virtue of various biochemical and physiological functions mediated by their metabolites [11]. There are four types of flora constituting the intestinal microbiota ecosystem: Firmicutes, Bacteroidetes, Proteobacteria, and Actinobacteria. Firmicutes and Bacteroidetes constitute a large proportion of the intestinal microbiota. The ratio of Firmicutes (F) and Bacteroidetes (B) (F/B) is regarded as an important biomarker for gut dysbiosis. Imbalances in the constitution of the intestinal microbiota (dysbiosis) have been linked to atherosclerosis, hypertension, heart failure, and other diseases [11]. Various factors, such as dietary habits, intestinal infection, and environmental factors, can change the balance of the intestinal microbial environment. Moreover, the intestinal microbiota can also produce trimethylamine (TMA) by metabolizing choline, phosphatidylcholine, and L-carnitine. The hepatic flavin monooxygenases (FMO3) enzyme then oxidizes TMA into trimethylamine N-oxide (TMAO). When TMAO is generated, several physiological processes can affect the host system by activating different signaling pathways. [11] Studies have shown that the elevation of TMAO can inhibit the transport of cholesterol and increase the accumulation of cholesterol in the macrophages, thereby accelerating the process of atherogenesis. Therefore, TMAO is pro-atherogenic, pro-thrombotic, and a contributor to ischemic heart disease; it is also linked to a bad prognosis in heart failure patients [11].

**Figure A1:**
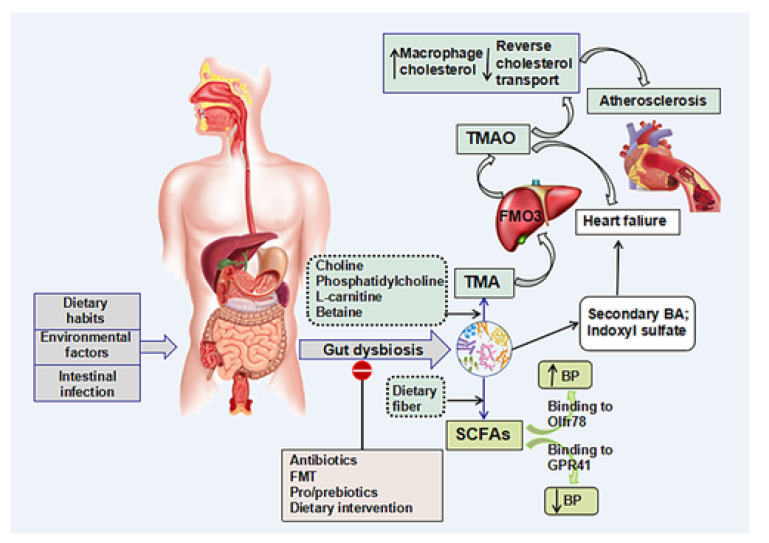
The role of intestinal microbiota in cardiovascular diseases. The figure is adapted with permission from 2019© Jin M. et al. [11].

Intestinal microbiota also generates some short-chain fatty acids (SCFAs) that attach to G protein-coupled receptor 41 (GPR41) and vascular olfactory receptor 78 (Olfr78), producing hypertensive and hypotensive effects, respectively. Thus, these metabolites act as a unique target for the management of hypertension. Studies found an association between intestinal microbiota-mediated inflammatory and immune responses and HF. According to the “gut microbiota” hypothesis, the reduced cardiac output leads to diminished tissue perfusion and, consequently, leads to intestinal ischemia in HF patients. Subsequently, this alters the constitution of the microbiota ecosystem. The levels of TMAO are prognostic of the long-term risk of mortality in patients with HF. Additionally, the intestinal microbiota also produces p-cresyl sulfate (PCS) and phenylacetylglutamine by metabolizing aromatic amino acids and glutamine conjugation, respectively, which can indicate the risk of cardiovascular events [11]. The role of intestinal microbiota in cardiovascular diseases is illustrated in Appendix A Figure A1.

## 2. Pharmacological Therapeutics in the Treatment of CVDs

Apart from certain lifestyle changes to modifiable risk factors, treatment of CVDs involves the use of different medications, which include angiotensin-converting enzyme, ACE, inhibitors (Lisinopril), Angiotensin receptor blockers, ARBs, Losartan, calcium channel blockers (Amlodipine), anticoagulants such as Warfarin, antiplatelets such as aspirin, beta receptor blockers such as Bisoprolol, antiarrhythmics such as Amiodarone, diuretics such as Furosemide, Digoxin, nitrates such as glyceryl trinitrate, and cholesterol-lowering medications such as statins. Because the main CVD therapeutic options (blood-pressure-lowering, lipid-lowering, and antiplatelet drugs) have largely independent mechanisms of action, fixed-dose combinations of these effective medicines have been promoted. Worldwide, poorly controlled blood pressure is responsible for the development of 62% of cerebrovascular and 49% of ischemic heart disease. Medications to lower serum lipids, such as statins (HMG-CoA reductase inhibitors), can decrease the risk of major cardiovascular events by 20%. Antiplatelet drugs, such as low-dose aspirin, have been reported to play an important role in the prevention of coronary artery disease and stroke [12] (Table 1).

## 3. Gut Microbiota: Link with Cardiovascular Diseases

Almost 100 trillion microbial cells (microbiota) are contained in the human gut, which are involved in the regulation, development, and proper functioning of mucosal barriers. The microbiota is significantly important in maintaining human well-being by regulating immunity, interfering with the colonization of pathogens, and synthesizing hormones and vitamins. Out of the total blood in our bodies, 40% is present in the gut. In CVDs, the gut is the primary body part that experiences ischemia and undergoes recovery at the end, during which the colonic microvilli and villi become susceptible to cell hypoxia and anaerobic metabolism [13]. All these factors ultimately lead to an unstable gut microbial composition (dysbiosis), which is mostly demonstrated by a reduction in *Bifidobacteria* and *Bacteroides* (Gram-positive bacteria) and a rise in *Proteobacteria* and *Firmicutes* (Gram-negative bacteria) [14]. The current research has recognized an association amid the gut microbiome and the pathophysiology of CVDs. Many studies have specified that microbial metabolites, as well as components present in the bacterial structure, can translocate from the intestine into the general circulation, thereby interacting and amending the functioning of metabolically pertinent tissues [15]. The gut microbiota produces TMAO, SCFAs, and bile acid that have several metabolic effects in humans. When there is gut dysbiosis during a diseased condition, injurious metabolites will intensify, which may be responsible for various pathologic processes of CVDs. The subsequent sections provide the details of these metabolites [16].

### 3.1. TMAO

Amongst different physiologically dynamic metabolites of the bacterial metabolism, TMAO has gathered substantial consideration as a factor responsible for CVDs. TMAO can gather in the kidney and heart, contributing to several biotic mechanisms, such as stimulating platelet accumulation, enhancing foam cell synthesis, and activating inflammatory responses [17]. Animal products, such as milk, red meat, and eggs, that contain a high amount of trimethylamine (TMA) moieties, such as L-carnitine and choline, are ingested to produce TMAO. TMA lyases metabolize these nutrients to synthesize TMA, and hepatic flavin monooxygenases oxidize these into TMAO, which further enters the systemic circulation and produces metabolic effects. In one of the meta-analyses (eight studies with 11,750 subjects), Ge et al. concluded that in comparison to reduced levels of TMAO, elevated levels of TMAO were associated with a higher risk and prevalence of hypertension [18]. The exact mechanism of how TMAO increases the risk of hypertension is still unknown.

### 3.2. SCFAs

Dietary fibers are fermented to produce SCFAs, which have appeared as significant signaling molecules with different ranges of physiological effects. The most plentiful SCFAs include propionate (C3), acetate (C2), and butyrate (C4) and encompass 95% of the total SCFAs present in the body. The concentration of these SCFAs in the intestine range from 10 Mm to 100 mM and produce several actions inside the gastrointestinal tract, such as the activation of ileum movement, mucus synthesis, and epithelial health protection (by upregulating the tight junction protein expression and as a main source of fuel for the epithelial cells present in the colon). Even though the large intestine metabolizes most of the SCFAs, a minor fraction becomes imbibed in the general circulation [19]. Numerous trials have revealed that SCFAs reaching general circulation modulate cardiovascular functions. For example, the supplementation of butyrate or acetate decreased the blood pressure (BP) in investigational hypertension models [20]. Roshanravan et al. recently found that the supplementation of butyrate for 45 days decreased systolic and diastolic pressure in diabetics [21].

### 3.3. Bile Acids (BA)

The liver synthesizes primary bile acids (BA), which are secreted into the small intestine to help in digesting and further absorbing the vitamins soluble in fat and lipids. Inside the intestine, secondary BA (e.g., lithocholic and deoxycholic acid) are formed by the intestinal microbiome [22]. It is also reported that the gut microbiota decreases the size and modifies the BA pool composition [23,24]. The association between microbiota and the BA seems to be bidirectional, as BA changes the microbial composition in the gut. In one study, it was reported that supplementation with cholic acid intensely transformed the microbiota in rats at both the phylum as well as class level, mostly resulting in a noticeable growth of Firmicutes (from 54% to 98%) at the expense of Bacteroidetes [25].

## 4. Gut Barrier Function and Bacterial Component Translocation

Several trials have related bacterial alterations of the gut with epithelial barrier function [26,27]. Factors, such as unevenness, amid colonic epithelial cell apoptosis and proliferation and decreased tight junction protein expressions, such as zonula occludens-1 (ZO-1) and claudin-1, can lead to intestinal permeability [28,29]. The barrier function must remain proper for reducing the intestinal content translocation, including the entry of bacterial components into the circulation. Due to disruption of barrier function, an immune response is stimulated by the pathogen-associated molecular patterns (PAMPs), leading to inflammation in the tissues. Thus, barrier function disruption due to dysbiosis is a major factor responsible for inflammatory processes and diseases linked to inflammation, such as obesity and diabetes [30,31]. Out of different microbial components recognized by the host’s immune receptors, lipopolysaccharide (LPS) and peptidoglycan have gained much consideration in association to CVD risk.

### 4.1. LPS

LPS, present in Gram-negative bacteria, are one of the major PAMPs studied in relation to CVDs. The innate immune responses are activated by the LPS via Toll-like receptor 4 (TLR4) signaling pathways. In short, there is LPS movement from the colon by either transcellular or paracellular pathways into the small intestine lymphatics or portal vein, individually [32,33]. As the LPS reach circulation, they rapidly bind to LPS-binding protein (LBP) synthesized in the liver [34], enabling LPS to attach to the TLR4 receptor on desired cells and muscles. The TLR4 stimulates an intracellular signaling cascade which results in the transportation of NF-ĸB and enhancement of many proinflammatory target genes [35]. A high-fat diet is responsible for LPS absorption across the intestinal barrier via assimilation inside chylomicrons and negotiated functioning of the gut barrier, respectively [36,37]. In one study, it was confirmed that inherently overweight mice, which were fed a high-fat diet, exhibited a two to three times enhancement in LPS, a range demarcated as “metabolic endotoxemia” [36,38]. The increased level of LPS resulted in gut dysbiosis, an enhancement of intestinal wall permeability, and a consistent decrease in tight junction proteins. Numerous studies have confirmed that LPS may lead to increased CVD risk. Initially, the LPS level is elevated in at-danger individuals and envisages imminent CVDs [39,40,41]. Second, vascular cell development due to LPS provokes a response parallel to the one detected throughout atherogenesis with oxidative stress, macrophage activation, cell death, inflammation, and adhesion of monocytes [42,43]. Finally, LPS supplementation at a reduced dose in animal models at the same concentration that is detected in endotoxemia leads to inflammation and atherosclerosis [43]. Together, these reports strongly suggest that LPS is a significant factor relating dysbiosis of the gut to CVDs.

### 4.2. Peptidoglycans

These are the chief components of the cell wall in the Gram-positive bacterial and negligible constituents in Gram-negative bacteria that may lead to CVDs. The fragments of peptidoglycan result in inflammatory gene transcription via MAPK and NF-κB signaling pathways [44]. Undeniably, peptidoglycans are detected inside atherosclerotic plaque cells in humans, which are linked with enhanced plaque inflammation [45].

## 5. Clinical-Trails and Animal Studies Demonstrated the Relationship between Dysbiosis and CVDs

Many trials have revealed a connection linking alterations in gut microbial composition and CVDs. Some of these clinical and animal studies are discussed below, and others are summarized in Table 2.

Some trials have proved that plasma levels of TMAO are a risk factor for CVDs [64]. Nonetheless, in some clinical trials, these raised levels of TMAO have been autonomously related to the incidence of CVDs and risks of stroke, myocardial infarction (MI), and death; thus, further study is required for recognizing the existing mechanism [64,65,66,67,68]. Additional research has revealed that a TMA-containing nutrient (L-carnitine) present almost entirely in red meat acts as a nourishing pioneer to gut synthesis of TMA and TMAO in humans and mice [65]. While betaine, choline, and TMAO were linked with an enhanced risk of CVD in 1876 patients through a heart risk assessment [64], additional studies in cohorts revealed that the prognostic importance was regularly limited to the formation of TMAO, particularly from L-carnitine and choline [65,69]. In a prospective human trial of greater than 4000 individuals taking coronary angiography, increased TMAO levels produced the main adverse effects on the heart, including stroke and MI for a duration of 3 years.

Studies of Metagenomics have established a positive association between TMAO formation and Clostridium species [65] and a positive connection between Clostridium histolyticum/perfringens and weight, waist circumference, and fat deposits [69]. Such works propose that this bacterial species, which is aforementioned, and *Atopobium* could be the inflammatory and CVD risk markers. Plasminogen activator inhibitor-1 (PAI-1) levels and BP are related to the intestinal microbiota composition in pregnant females who are overweight. The abundance of the butyrate-synthesizing genus *Odoribacter* has remained contrary in connection with systolic BP. The capacity of butyrate synthesis is minor, and PAI-1 levels are higher in overweight pregnant females. Additionally, the levels of PAI-1 are also equally associated with the expression of butyrate kinase and the richness of the *Odoribacter* species [70,71].

Three current meta-analyses have recognized that raised plasma levels of TMAO are linked with enhanced risks of CVD and all-cause death [72,73,74]; yet, some condemnation occurs about the CVD and TMAO association as fish might hold larger TMAO and TMA concentrations [71]. Nevertheless, the intake of fish is good for cardiac health [75,76,77]. Similarly, there is a trial with no association between events of TMAO and atherosclerosis [78]. More randomized human studies with an increased number of subjects are required to elucidate if TMAO is a mediator or marker in CVD.

One of the studies, which included three different male groups and was published in accordance with the European Society of Hypertension (ESH) criteria for hypertension, found relationship between increased SCFA levels and lower (improved) BP readings [79].

It has been pragmatic how the count and type of bacterial species present in the buccal cavity may be associated with CVDs [80]. A trial in individuals hospitalized for acute coronary syndrome exhibited an increased subgingival concentration of bacteria in comparison to healthy individuals; the classes that were frequently enhanced in the trial include *Tannerella forsythensis, Porphyromonas gingivalis Streptococcus intermedius*, and *S. anginosu*. Henceforth, such types can be the factors responsible for the progression of the acute coronary condition [81]. Moreover, a likely connotation between *Actinobacillus actinomycetemcomitans* existing in the buccal cavity and both stroke and coronary artery disease (CAD) has been defined after numerous sero-epidemiologic trials [82,83].

A diet rich in choline enhanced the levels of TMAO and atherosclerosis in mice, which was contingent on the activity of gut microbiota, as revealed by a comprehensive range of antibiotic treatments [84]. One more study on male subjects with atherosclerotic plaque who consumed a beverage containing a higher quantity of *Lactobacillus plantarum* (DSM9843) revealed an enhanced microbial assortment as well as a reduction in the quantity of SCFA levels [85], which signifies that the intake of this species may be an approach favoring the colonic variety in subjects with atherosclerotic plaque.

**Figure A2:**
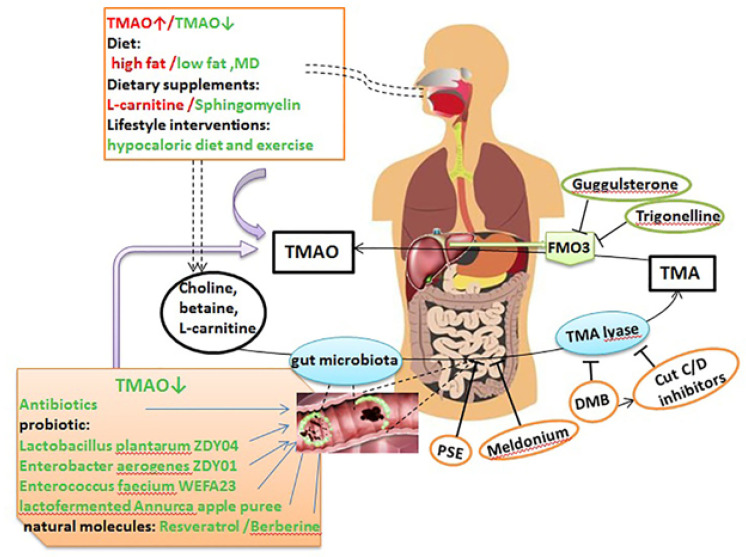
TMAO potential as a therapeutic target in atherosclerosis. This figure is adapted with permission from: 2019©Yang S, et al. [84].

Current trials have linked increased TMAO concentrations to an enhancement of CVD risk and its sternness [86,87]. Consequently, TMAO concentration is connected with the size of atherosclerotic plaque and CVD proceedings [64] (Appendix A Figure A2).

Additional investigational works have revealed that such plaques comprise bacteriological DNA, and the bacterial taxa seen have also existed in the gut and oral microbiome of similar subjects [88,89]. Numerous epidemiological trials have linked gum disease and diseases of the tissues surrounding teeth with CVD [90,91,92]; an oral microbial role in the pathogenesis of CVD has been also considered [92,93,94].

Additionally, metagenomic studies have revealed that the bacterial arrangement is changed in subjects who are having unbalanced plagues in comparison to constant plaques. The unbalanced plaques are associated with decreased fecal concentrations of *Roseburia* together with an enhanced hypothetical volume of the microbial species to synthesize proinflammatory peptidoglycans and decrease the synthesis of anti-inflammatory carotenes [95].

Additional cases correlating atherosclerosis and microbiota include the supplementation of whole grains and metformin. Metformin is extensively utilized in people who have revealed positive actions that compete against the danger of CVD. Additionally, it may be securely utilized in subjects with cardiac failure and may also directly decrease its incidence or attributable mortality [96] likely due to actions produced via remodeling of the gut microbiota [96]. Likewise, food appears to be a possible treatment to reduce the risk of CVDs. For example, a trial on precise inhabitants of Danish adults presented that food rich in whole grains rather than refined grain decreases the major risk factors (systemic inflammation and body mass) related to injurious projection of CVDs [97]. Mechanisms of how the intestinal microbiota may affect cardiovascular drug outcomes are given in Table 3.

## 6. Therapeutic Gut-Microbiome Interaction

The intestinal microbiota and their metabolites have become potential therapeutic targets for certain antibiotics, probiotics, prebiotics, dietary interventions, and fecal microbiota transplantation (FMT) [11].

### 6.1. Antibiotics

Oral antibiotics, such as neomycin, minocycline, and vancomycin, act by changing the balance of the intestinal microbiome environment and thus are found to regulate the blood pressure. Ampicillins could decrease low-density lipoprotein (LDL) and very-low-density lipoproteins (VLDL) cholesterol levels. Since these antibiotics have been shown to cause a broad range of side effects, their potential advantages versus health risks need to be determined [11].

### 6.2. Fecal Microbiota Transplantation (FMT)

The use of beneficial bacteria in the form of fecal microbiota transplantation (FMT) from healthy individuals inhibits the multiplication of harmful bacteria and modifies the host’s immune system, assisting patients in restoring normal intestinal microbiota functions [102]. FMT is found to be efficacious in the management of infections caused by Clostridium difficile, irritable bowel syndrome, and recurrent inflammatory bowel disease. FMT can be a potential treatment for CVDs; however, more studies are needed to be done to check its efficacy [11].

### 6.3. Probiotics and Prebiotics

Probiotics, such as *Lactobacillus, Bifidobacterium, Lactococcus,* and *Streptococcus,* can stimulate the growth and activity of certain intestinal microbiota, which can regulate the immune response and limit the extent of inflammation, thereby benefiting the host. Prebiotics are complex carbohydrates that are found in foods such as peas, beans, whole grains, and cereals. They can also improve gut dysbiosis, which has beneficial implications in the treatment of CVDs [11]. A recent animal study found that a regular intake of probiotics has been linked to the successful reduction and control of blood pressure (BP) among rats with hypertension and delayed the development of heart failure following myocardial infarction. It has been hypothesized that BP reduction could be mediated by the effects of probiotics on the autonomic nervous system, primarily through the interaction between gut microbiomes and the nervous system via afferent sensory neurons in the intestine [103,104,105]. In one randomized controlled clinical trial, it was seen that prebiotic inulin, when combined with probiotic strain *Lactobacillus Rhamnosus G,* led to a remarkable reduction in inflammatory biomarkers, such as TNF-α and LPS, and depression in patients suffering from coronary artery disease. Moreover, the effect was more significant when both inulin and the probiotic were given in combination as compared to when both were given individually [106]. Accruing evidence reveals that probiotics decrease LDL-cholesterol, improve the LDL/HDL ratio, and have the potential to reduce blood pressure, body mass index, inflammatory markers and normalize the blood sugar level. Consequently, these factors increase their scope of use to be industrialized as nutritional supplements, likely with cardiac health benefits. Nevertheless, there is not only uncertainty concerning the precise dosages and strains of the probiotics that will provide beneficial health effects but also issues, such as genetics as well as immunity of the person, that may affect the effectiveness of the probiotics. Therefore, additional experiments and research are necessary to better understand the mechanism of probiotics as well as rule out any of their feasible side effects [107].

### 6.4. Dietary Intervention

Studies have shown that a diet enriched with acetate and/or a high-fiber diet leads to an alteration of the gut microbiota and increased levels of SCFAs, which have a positive impact on patients with HF and hypertension [11]. SCFAs, such as acetate and butyrate, which are generated by the bacterial fermentation of dietary fiber, are absorbed from the colon. They improve dysfunction of the endothelial wall caused by the prohypertensive agent angiotensin-II by elevating the availability of nitric oxide, which is involved in the activation of G protein-coupled receptors (GPR41 and GPR43) [108]. In addition, resveratrol that is found in grapes and berries act by inhibiting TMA and thus has favorable effects on atherosclerosis [109]. Thus, dietary changes may delay the development of CVDs.

### 6.5. Aspirin and Gut Microbiota

Aspirin is one of the non-steroidal anti-inflammatory drugs (NSAIDS) that has been widely used to lower the risk of cardiovascular and cerebrovascular diseases [110]. Individuals consuming aspirin presented with different gut microbiota profiles than non-users demonstrated the aspirin’s ability to alter the gut microbiota composition. For instance, patients who are on aspirin tend to have a change in the gut microbiota composition, including changes in the proportion of *Prevotella, Ruminococcaceae, Bacteroides*, and *Barnesiella* bacteria when compared to non-users or other NSAID users. While only a few studies have shown the effects of the gut microbiota on the absorption, metabolism, distribution, and efficacy of NSAIDs, several other reports have indicated that an alteration in the composition of the gut microbiota can influence the metabolism of aspirin. For example, the administration of oral antibiotics can reduce the metabolic activity of the gut microbiota, thus slowing the host degradation of the orally ingested aspirin, which increases its bioavailability and prolongs its anti-thrombotic effects [111]. Conversely, the ingestion of probiotics rich in *Bifidobacteriumbreve Bif195* bacteria can protect from intestinal wall damage and gastric ulcers induced by aspirin intake [112].

### 6.6. The Effects of Beta Blockers, ACEi, and ARBs on Gut Microbiota

The effects of antihypertensive medications, such as beta-blockers, ACE inhibitors, and ARBs, have been studied in humans and animals. Reports from human cohort studies showed an association between the use of beta-blockers, ACE inhibitors, and ARBs and the imbalance in the composition of the gut microbiota environment. These findings may oppose what is expected when initiating anti-hypertensive therapy, as one would expect that they would improve the gut dysbiosis. A large metagenomic study of 1135 participants found a positive correlation between ACE inhibitors and calcium channel blockers and the gut microbiota composition. Surprisingly, a study on rats found that spontaneously hypertensive rats treated with ACE inhibitors, such as captopril, experienced a reduction in gut dysbiosis, improved permeability of the intestinal wall, and increased villi length [98]. This highlights the importance of further large analyses to better understand the influence of anti-hypertensive medications on the gut microbiota composition in humans.

### 6.7. Statin and Gut Microbiota

Simvastatin, atorvastatin, and rosuvastatin are the three most commonly used statins to lower cholesterol and low-density-lipoproteins-C (LDL-C). Recent studies have shown that statins can modulate the composition of the gut microbiome [98]. In a cross-sectional study that assessed the microbiota composition in 15 untreated patients with hypercholesterolemia and 27 atorvastatin-treated hypercholesterolemia patients. The group of patients who were treated with atorvastatin displayed an abundance of anti-inflammatory microbiota gut species, including *Fecalibacterium prausnitzii* and *Akermansia muciniphlia*, while patients who were not treated with atorvastatin displayed bacterial species associated with inflammation, including *Collinsella* and *Streptococcus* [113].

### 6.8. Other Therapies

Studies have also shown that certain analogs of choline, 3, 3-dimethyl-1-butanol act by inhibiting Trimethylamine (TMA) N-oxide (TMAO) formation and thus can reduce atherosclerotic lesions [114]. Bacterial fermentation of dietary choline, L-carnitine, and betaine produces trimethylamine (TMA), which gets metabolized into trimethylamine oxide (TMAO) by gut flora and is then metabolized into TMAO by the hepatic enzyme flavin monooxygenase-3 (FMO3) and eventually excreted in the urine [115]. There is increasing evidence that the FMO3 enzyme, its substrate (TMA), and the product (TMAO) have been linked to a number of various conditions, such as cardiovascular disease, reversing cholesterol transport, and the metabolism of glucose and lipids. Moreover, FMO3 is also primarily responsible for the metabolism of various therapeutic molecules. This, together with TMA, indicates that dysbiosis could affect the absorption and thus the efficacy of therapeutic drugs [115]. Thus, the inhibition of the several steps involved in the production of TMAO can lead to reduced levels of TMAO and hence can be used as potential targets for the management and prevention of atherosclerosis [84]. Dietary compounds, such as choline, betaine, and L-carnitine, are the main precursors of TMAO production, which are degraded by the intestinal microbiome and their derived enzymes to produce TMA. TMA is then absorbed and transported to the liver, where it can be converted by FMO3 into TMAO. Several pathways can be used to regulate the production of TMAO, such as via lifestyle changes, including diet and the consumption of dietary supplements, antibiotics, prebiotics, probiotics, and other naturally occurring molecules, all of which can decrease TMA and TMAO levels by remodeling the gut flora. Meldonium, DMB, PSE, and CutC/D inhibitors can inhibit the production of TMA, while guggulsterone and trigonelline can inhibit the metabolism of TMA into TMAO by inhibiting FMO3 [84]. Methimazole and indole act by inhibiting FMO3 and thus can be used to prevent and treat atherosclerosis [11].

## 7. Clinical Applications of the Drug-Gut Interactions

As we learn more about the scope and clinical relevance of drug–microbiota interaction, we realize that the gut microbiota not only affects drug absorption and distribution but can also metabolize drugs, thus altering their efficacy. The drug-induced alteration in the microbial composition and the microbial-induced alteration in the drug absorption and distribution have critical effects on the host system and the health outcome. We now understand that some anti-inflammatory medications, anti-hypertensive medications, and lipid-lowering medications can interact with the gut environment and have a positive or negative association with the microbiota composition. This raises awareness that when treating cardiovascular disease patients with these drugs, they need to be on probiotics or prebiotics or placed on special dietary and lifestyle changes [116]. It also highlights the effects of polypharmacy on the gut microbiota composition, and thus, providers should continuously monitor drug-associated effects in vulnerable patients where the microbiota ecosystem is already compromised. One approach to achieve that is through sequencing patient fecal samples, which serve as a proxy for the gut microbial composition, and then marking the absence or presence of a particular microbe or enzyme. Using this technique combined with a machine learning algorithm, one can predict drug safety, efficacy, and metabolism. Moreover, highlighting the current view of drug–microbial interactions creates a better understanding of internal factors that shape drug concentration and toxicity, which can affect the appropriate dose measurement per patient per condition [117].

Lastly, there is an increasing amount of evidence about the role of gut microbiota in predicting the prognosis and outcomes of cardiovascular diseases. Therefore, it is now regarded as a reliable target for disease management and prevention. Maintaining a balanced microbiota environment has been linked to improved lipid profiles, blood pressure measurements, and improved BMI among patients with metabolic syndromes. Therefore, it is intuitive when treating patients with cardiovascular diseases to also target the gut microbial environment through dietary interventions to amplify treatment outcomes [118].

## 8. Conclusions

Dysbiosis is an imbalance in the composition of the intestinal microbial environment that alters the ratio of Firmicutes (F) to Bacteroidetes (B) (F/B). The recent findings on the role of intestinal microbiota and its implications in CVDs and its risk factors have highlighted the critical link between the heart and the gut. Despite these intriguing and notable results, by comparison, few studies have demonstrated evidence on the implications of CVD therapies on the composition and balance of the intestinal microbial ecosystem in patients with CVDs. Moreover, we aimed to gain a better understanding of the drug-to-microbiome interaction and microbiome-to-host interaction and their association to underlying molecules that play a role in disease susceptibility and disease outcome. Studies aimed at revealing these sorts of details are highly warranted to be able to leverage knowledge gained to develop therapeutic interventions.

## Figures and Tables

**Table 1:** List of drugs included in the WHO Model List of Essential Medicines (EML).

Therapeutic Group According to EML	International Non-Proprietary Name (INN)
Angiotensin converting enzyme inhibitors	Lisinopril
Anticoagulants	Heparin, Warfarin
Antithrombotic Agents	Streptokinase
Antiarrthymics	Lidocaine, Amiodarone
Arteriolar dilator	Hydralazine
Beta adrenergic receptor blockers	Bisoprolol, Carvedilol
Beta Lactam Antibacterials ^†^	Benzathine benzylpenicillin, Phenoxymethylpenicillin
Cardiac glycosides	Digoxin
Cardioselective calcium channel blockers	Verapamil
Centrally acting sympatholytics	Methyldopa ^†^
High-efficacy diuretics	Furosemide
Insulins and oral antidiabetic medications	Insulin injection, Intermediate-acting insulin, Glicazide, Glucagon Metformin
Lipid modifying agents	Simvastatin
Low efficacy diuretics	Hydrochlorothiazide
Organic nitrates	Glyceryl Trinitrate, Isosorbide Dinitrate
Other analgesics and antipyretics	Acetylsalicylic acid
Positive inotropes except digoxin	Dopamine, Adrenaline
Potassium sparing diuretics	Spironolactone
Vasoselective calcium channel blockers	Amlodipine

Notes: ^†^ Methyldopa is listed for use in the management of pregnancy-induced hypertension only. Its use in the treatment of essential hypertension is not recommended in view of the availability of more evidence of efficacy and safety of other medicines. For prevention of rheumatic heart disease; we listed those medicines because they are included in the “best buy” strategies of the World Health Organization to reduce cardiovascular diseases. This paper focuses on access to medicines for CVD and not specifically to these medicines.

**Table 2:** Summary of trials explored connections between gut microbiota and CVDs.

Clinical Studies				
CVD	No. of Patients	Change in Gut Microbiota Composition/Metabolites	Outcome	Reference
Atherosclerosis	332	Increased LBP	Increased carotid intima media thickness	[46]
	4144	Increased TMAO	Increased atherosclerotic risk	[47]
CAD	2255		Increased risk of artery infarction	[48]
	59	Increased L-carnitine	Increased TMAO in CAD patients	[49]
	126	Increased LPS	Increased inflammatory cytokines	[50]
	30	Reduced *Bacteroides vulgatus* and *B. dorei* and LPS	Increased lesions	[51]
CAD and artery stenosis	169	Increased TMAO	Increased risk of CAD and artery stenosis	[17]
Heart failure	122	Increased LPS	LPS translocation through leaky gut, resulting in inflammation	[52]
	452		Endotoxemia inflammation and oxidative stress	[52]
Heart attack	38	Increased proteobacteria LPS and leaky gut	Increased endotoxemia	[53]
Atrial fibrillation	912	Increased LPS	Increased platelet activation	[54]
**Animal studies**				
**CVD**	**Animal**	**Change in gut microbiota composition/metabolites**	**Outcome**	**Reference**
Atherosclerosis	Mice	Increased LPS	Activation of NF-κB and JNK pathways	[55]
	Mice		Increased size of atherosclerotic lesions	[56]
	Mice		Increased proinflammatory cytokines	[57]
	Mice	Increased TMAO	Nlrp3 inflammasome stimulation and endothelial dysfunction	[58]
	Mice		Increased plague area	[48]
			Increased expression of inflammatory genes	[48]
	Mice	Butyrate supplementation	Reduced cholesterol absorption and atherosclerotic lesion	[59]
	Mice	Reduced SCFAs and *Akkermansia*, *Clostridium*, and *Odoribacter*	Increased plague size	[60]
	Mice	Reduced *Bacteroidetes* and *Clostridia*	Increased dyslipidaemia	[61]
Heart failure	Mice	Increased TMAO	Increased severity of heart failure	[48]
Hypertension	Rat		Increased osmotic pressure and water reabsorption	[62]
Cardiomyopathy	Mice	Increased LPS	Increased inflammatory markers	[63]

CAD: Coronary artery disease; LPS: Lipopolysaccharides; TMAO: Trimethylamine N-oxide; LBP: Lipopolysaccharide binding protein; SCFAs: Short chain fatty acids; NF-κB: Nuclear factor kappa beta.

**Table 3:** Mechanisms by which the gut microbiota may influence cardiovascular drug outcomes.

Drug	Bacteria	Mechanism(s)	Outcome	Reference
**Known microbiome–drug interactions**				
Digoxin	*Eggerthella lenta*	Inactivation by reduction	Bacterial reductase activity decreases the amount of the active drug reaching target tissues	[98]
**Proposed microbiome–drug interactions**				
Simvastatin	Not known	Microbial-derived bile acids competing for host uptake transporters Alteration in bacterial communities with *bile salt hydrolase (bsh)* activity	Decreased amount of the drug reaching target tissues Variability in FXR receptor signaling	[98,99]
Rosuvastatin	Not known	Alteration in host gene expression in bile acid metabolism pathways Alteration in bacterial communities with *bile salt hydrolase (bsh)* activity	Variability in FXR receptor signaling	[98]
Atorvastatin	Not known	Decreased amount of secondary bile acids	Variability in FXR receptor signaling	[98]
Lovastatin	Not known	Increase metabolism (hydrolysis)	Altering its lipid lowering activity	[100]
Amlodipine	Not known	Pre-systemic metabolism by dehydrogenation	Decreased amount of the active drug reaching target tissues	[98]
Nifedipine	Not known	Decreased absorption	Decreased potency of the drug	[101]
Captopril	Not known	Not known	Decreased intestinal permeability and improved villi length	[98]
Enalapril	Not known	De-esterification	Increased biotransformation	[101]
Aspirin	Not known	Hypoxic conditions alter the metabolic activity of the intestinal flora	Increase absorption of aspirin in rats and increased risk of bleeding	[101]
Amiodarone	*Escherichia coli* Nissle *1917*	Not known	Increased activity	[100]
Glyceryl trinitrate		Denitration	Decreased activity	[100]
Quercetin-3-glucoside	*Eubacterium ramulus* and *Enterococcus casselilfavus*	Deglycosylation	Decreased activity	[100]
**FXR—farnesoid X receptor**
